# Prevalence of cutaneous leishmaniasis infection and clinico-epidemiological patterns among military personnel in Mullaitivu and Kilinochchi districts of the Northern Province, early war-torn areas in Sri Lanka

**DOI:** 10.1186/s13071-020-04137-8

**Published:** 2020-05-19

**Authors:** Nayana Gunathilaka, Saveen Semege, Nishantha Pathirana, Nuwani Manamperi, Lahiru Udayanga, Harshima Wijesinghe, Prasad Premaratne, Deepika Fernando

**Affiliations:** 1 Department of Parasitology, Faculty of Medicine, University of Kelaniya, Ragama, Sri Lanka; 2 Directorate of Army Preventive Medicine & Mental Health Services, Army Headquarters, Sri Jayawardenepura, Sri Lanka; 3 Sri Lanka Army Hospital, Colombo, Sri Lanka; 4 Department of Biosystems Engineering, Faculty of Agriculture and Plantation Management, Wayamba University of Sri Lanka, Makandura, Sri Lanka; 5 Department of Pathology, Faculty of Medicine, University of Colombo, Colombo, Sri Lanka; 6 Department of Parasitology, Faculty of Medicine, Kotalawela Defence University, Ratmalana, Sri Lanka; 7 Department of Parasitology, Faculty of Medicine, University of Colombo, Colombo, Sri Lanka

**Keywords:** Cutaneous leishmaniasis, Clinical, Epidemiological, War-torn areas

## Abstract

**Background:**

The 30-year-old armed conflict in Sri Lanka resulted in a general breakdown of civil administration in the Northern and Eastern provinces, leading to mobilisation of many armed forces personnel to assist with reconstruction and resettlement. This occupational group has been identified as a priority risk group for leishmaniasis.

**Methods:**

Individuals enlisted at all military establishments in Mullaitivu and Kilinochchi districts, Northern Province of Sri Lanka were included. Five thousand individuals were screened for skin lesions between September 2018 and August 2019. Persons with lesions suspected as cutaneous leishmaniasis (CL) were further investigated. Information on sociodemographic/other potential risk factors was obtained through an interviewer-administered structured questionnaire. The diagnosis was confirmed by microscopic visualization of parasitic stages from different samples obtained (skin scraping, lesion aspirate and tissue impression smears), histopathology and polymerase chain reaction DNA amplification.

**Results:**

Among 5000 individuals screened, 74 individuals were suspected of having CL. Of these, 67.6% (*n* = 50) patients were confirmed for CL by microscopy. Around two third of both males (67.6%; *n* = 48) and females (66.6%; *n* = 2) were positive for *Leishmania*. The soldiers belonging to 26–35-year age group reported the highest susceptibility (83.3%; OR: 4.83, 95% CI: 3.49–6.20%). Of the sociodemographic factors, age, wearing short-sleeved upper body clothing as the uniform and non-use of insect repellents were identified as significant risk factors. Most of the CL patients had a single lesion (86.0%; *n* = 43) of an ulcerative type (34.0%; *n* = 17), mostly on their upper limb (67.9%; *n* = 34). Lesions were mostly 5–10 mm diameter (59.9%; *n* = 30) in size with poorly defined margins (72.0%; *n* = 36). Amongst the diagnostic techniques, microscopic examination of slit skin smear and tissue impression smear were able to discriminate the majority of patients (92.1%; *n* = 46) for CL.

**Conclusions:**

In order to highlight the true burden of leishmaniasis in the military personnel, cases of leishmaniasis from military institutes should be recognized as a different entity per say and be included in the national figures so as to depict the real magnitude of the disease burden amongst this high-risk group.
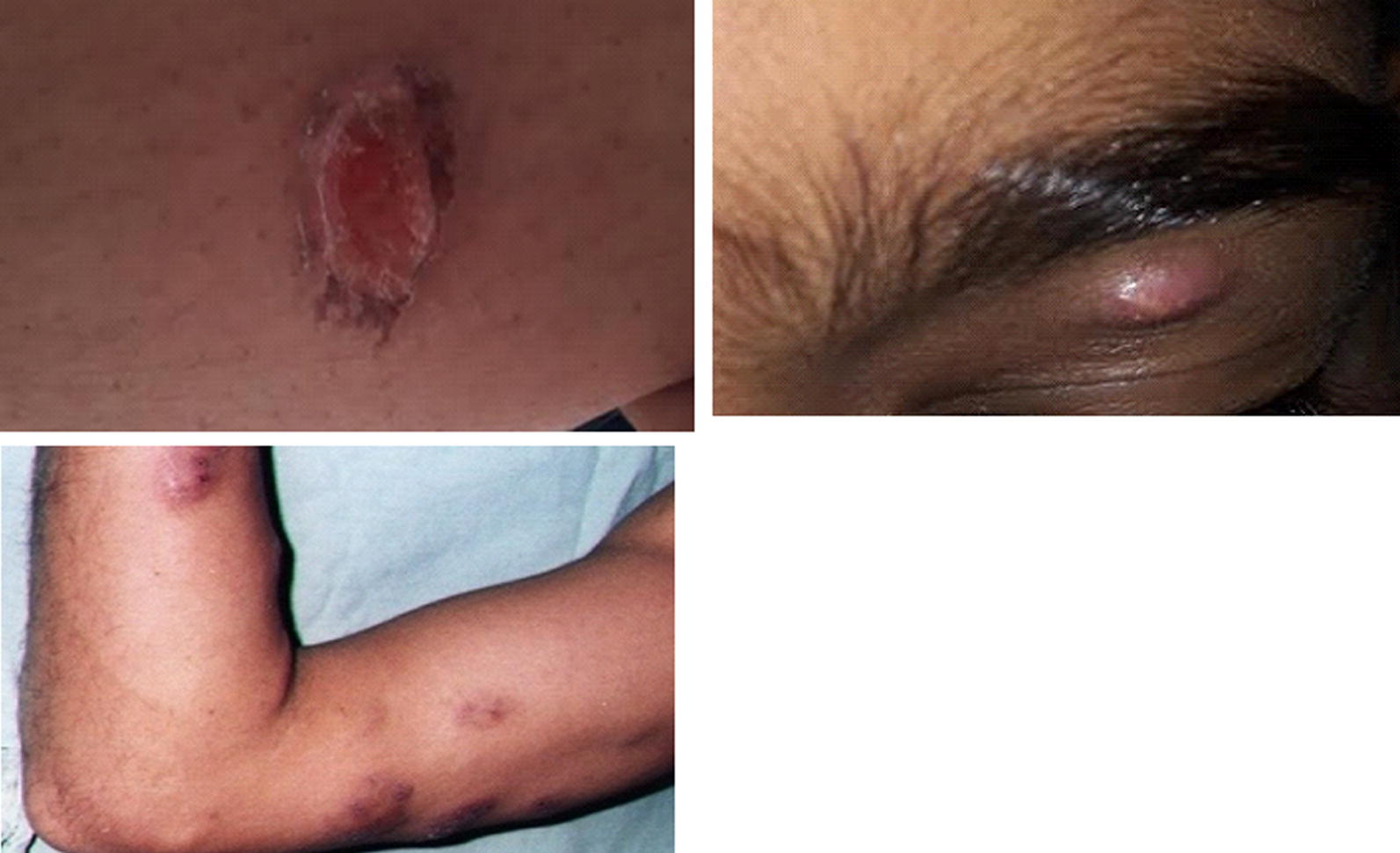

## Background

Leishmaniasis is one of the neglected tropical diseases in the world. The estimated global burden of this disease is believed to be higher than the reported numbers due to under-reporting, under-surveillance and inadequate case detection techniques [1]. The geographical distribution of leishmaniasis has expanded in the world significantly, with a concomitant sharp increase in the number of cases and emerging new disease foci [2, 3]. Several countries have experienced epidemics including the South Asian countries of Nepal, Bangladesh and India [4, 5].

The first autochthonous case of cutaneous leishmaniasis (CL) in Sri Lanka was reported from the Southern Province in 1992 [6]. A decade later, the disease remained sporadic with a few reported cases from various parts of the island [7, 8]. Case histories of these latter patients were compatible with the local acquisition of the disease and authors speculated an existing zoonotic cycle [8–10]. However, since 2002 the number of CL cases has increased mainly due to the influx of CL cases reported among military personnel who were serving in conflict areas in the northern part of the country [11].

The free movement of affected individuals between conflict-free areas of the country with already existing vector populations may have subsequently led to the spread of this disease more widely within the country. It is mostly the cutaneous form of the disease that has been reported from Sri Lanka, with three cases of endogenous visceral leishmaniasis (VL) and a few cases of endogenous mucosal leishmaniasis (ML) being reported [12–16]. The causative parasite strain responsible for both CL and VL in Sri Lanka has been identified by multilocus enzyme electrophoresis as *Leishmania donovani* MON-37 [17, 18].

Although the vector of leishmaniasis is yet to be confirmed, the existence of *Phlebotomus argentipes*, the major vector of VL in India has been reported in Sri Lanka [19–22]. A recent DNA analytical study had shown the existence of *L. donovani* DNA in a Sri Lankan *P. argentipes* population [23, 24].

The 30-year-old armed conflict in Sri Lanka resulted in a general breakdown of civil administration in the Northern and Eastern provinces. Mullaitivu and Kilinochchi districts were the front lines of conflict between the Sri Lankan armed forces and the separatists, bearing the brunt of the war which resulted in widespread destruction to public and private property as well as extensive displacement of people. In addition to indigenous transmission of leishmaniasis, following the end of the conflict in 2009 there has been a return of individuals who fled the country to South India, which still reports one of the highest numbers of leishmaniasis cases. These individuals, along with manual workers entering the country from India both legally and illegally, may form a reservoir of infection. Following the end of the conflict, the government initiated an intensive programme of resettlement of displaced populations, coupled with programmes to rebuild roads, houses and hospitals, which may have contributed to the establishment of new breeding sites. Further, a large number of armed forces personnel from all parts of the country have been mobilised to the Northern and Eastern provinces to assist with reconstruction and resettlement [25].

In 2011, it was reported that approximately 267 cases of CL had been diagnosed clinically over the preceding 26 months amongst an armed forces population of 15,000 involved in the rebuilding processes carried out in the districts of Mullaitivu and Kilinochchi (Sri Lanka Army Medical Services, personal communication). A preliminary active case detection survey carried out with limited funds amongst 4321 military personnel attached to one division in the Mullaitivu District indicated a prevalence rate of 0.58% (25 positive cases) by slit skin smear examination [26]. This indicates that transmission of the disease is occurring in the area.

At the time of carrying out this study, proper diagnostic facilities and social awareness programmes for leishmaniasis were not available in the military camps or the general hospitals of Kilinochchi and Mullaitivu. Therefore, the accurate number of leishmaniasis cases in the post-conflict districts remained undetermined. Hence, the present study aimed to determine the prevalence and study the clinico-epidemiological nature of leishmaniasis among military personnel currently working in the Kilinochchi and Mullaitivu districts which are post-conflict districts in Sri Lanka.

## Methods

### Study design and population

A prospective cross-sectional survey was conducted selecting the military personnel residing in all military establishments located in the Mullaitivu and Kilinochchi districts of the Northern Province to estimate the prevalence of CL. There are three military establishments in each district, housing approximately 5000 soldiers at each establishment, giving rise to a total of 15,000 military personnel in each district.

### Sample size calculation

Taking 0.5% as the expected frequency of the factor under study and 0.25% as the worst acceptable result, using EPI INFO statistical program [27], a population of approximately 5000 persons were estimated to be screened. Calculation of sample size was based on the following formula [28] where *n* = z^2^ p (100 − p)/d^2^. The *z*-value corresponding to the required level of confidence was taken at 1.96, the expected prevalence of leishmaniasis was taken as 0.5% and the desired level of precision was taken as 0.25%. As the systematic sampling was performed within each camp, a design effect of 1.5 was considered to minimize the clustering effect giving rise to a sample size of 4586. After allowing for 5% non-response rate the required sample size was defined as 5000. Systematic sampling was used to screen the study population. All the camps in each district were listed with the number of personnel in each camp. The sample size from each camp was decided proportionate to the number of personnel in the camp. According to the sample size of each camp, the sampling interval for systematic sampling was decided. The selection of participants was handled by the non-military investigators in the study group and was based on random selection. Individuals who refused to participate were replaced with new recruits. The information of the individuals was kept strictly confidential.

### Screening of the study population

Selected military personnel were screened between September 2018 and August 2019 by trained Army Public Health Inspectors. Persons were suspected with a diagnosis of CL based on the duration, examination findings and comparison of a skin lesion with the pictures on a colour plate, which was developed with the assistance of three experts in the field. Individuals suspected for CL were then referred to the Dermatology Clinic at the Army Hospital in Colombo for further evaluation. Information on sociodemographic/other potential risk factors and protective measures against vector biting was obtained through an interviewer-administered structured questionnaire.

### Clinical examination

Patients suspected of having CL were examined by a consultant dermatologist. Clinical details of the lesions (site of lesion/s, number of lesions, type of lesion, size, shape, consistency, scaling, itching, pain, pus/other discharge, crusting, evidence of secondary infection and skin changes) were collected through an interviewer-administered questionnaire. Previous attempts for medication were also recorded. Slit skin smear, fine-needle aspiration biopsy and tissue impression smear from each suspected patient were taken by a trained medical officer under the guidance of the consultant dermatologist.

### Preparation of smears

#### Slit skin smear (SSS)

After cleaning the lesion with 70% ethanol the edges of the lesion were compressed between two fingers and a nick 4–5 mm in length and 2–3 mm in depth was made at the active edge of the lesion with a scalpel blade (size 11). The gaped walls of the nick were scraped with the blunt side of the scalpel blade and a thin skin smear was prepared. If the lesion had a scab, it was removed, and tissue material was scraped from the base of the lesion [3] to prepare the smear.

#### Lesion aspirate (LA)

Smears from fine-needle aspirates were obtained by injecting 0.1–0.2 ml sterile saline into the edge of the lesion with a 23G needle fixed to a 2 ml syringe and applying suction while performing rotator to and for movement.

#### Tissue impression smear (TIS)

Two standard 2 mm diameter punch biopsy samples were collected from the active edge of the clinically suspected lesion under local anaesthesia (1% lidocaine hydrochloride) under sterile conditions. Each biopsy specimen taken for TIS was grasped gently with forceps and excess blood removed by placing it on a gauze towel. The specimen was rolled over the slide gently and air-dried.

### Parasitological examination

The slides prepared from skin scrapes LA and TIS were air-dried, fixed in methanol, stained with Giemsa and examined under oil immersion using a microscope. Detection of the oval-shaped amastigotes with the characteristic nucleus and kinetoplast (dot and dash appearance) was considered as diagnostic. The slides were checked by a panel of experts including two senior technical officers and two parasitologists at the Department of Parasitology, Faculty of Medicine, University of Kelaniya, Sri Lanka. Further, as a quality control measure, the slides were cross-checked by a consultant parasitologist at a reference laboratory to confirm the interpretation.

### Histopathological examination

Each 2 mm diameter punch biopsy sample collected from the active edge of the clinically suspected lesion under local anaesthesia was stored in 10% formalin for histopathology confirmation. The tissue samples were dehydrated, cleared, embedded in paraffin, cut into 4–5 μm thick sections and stained with hematoxylin-eosin (HE) and Giemsa. A positive control was performed with all cases stained with Giemsa stain. The biopsies were evaluated by two consultant histopathologists at the Department of Pathology, Faculty of Medicine, Colombo, Sri Lanka. The slides were viewed under a multihead microscope by the two pathologists and a consensus decision was made regarding the presence or absence of organisms. Positive and negative controls were available for comparison.

### Molecular diagnosis

#### DNA extraction and amplification

Skin scraping obtained from the suspicious lesion was introduced to a microcentrifuge tube filled with 500 μl of saline. The samples were kept at − 20 °C until DNA was extracted. The genomic DNA was extracted using the QIAamp DNA Mini Kit (Qiagen GmbH, Hilden, Germany) according to the manufacture’s guidelines.

Primers designed by Salora et al. [29] in a previous study were used for amplification (F: 5′-AAA TCG GCT CCG AGG CGG GAA AC-3′; R: 5′-GGT ACA CTC TAT CAG TAG CAC-3′) targeting kinetoplast mini-circle sequence (591 bp) of *L. donovani*. The amplifications were conducted using 20 μl of solution containing, 1 μl of DNA product as the template, 2 μl of CoralLoad Buffer (Qiagen) with 15 mM MgCl_2_ and loading dye, 1.6 μl of dNTP mixture with 0.2 mM from each nucleotide, 0.06 μl of 0.3 μM forward and reverse primers, 0.41 μl of 2.5 U DNA polymerase and 15.18 μl of PCR grade water. Amplification was performed in a thermal cycler (SimpliAmp™; Applied Biosystems, Thermo Fisher Scientific, USA) programmed for 30 cycles of denaturation at 94 °C for 30 s, annealing at 50 °C for 60 s, and extension at 72 °C for 24 s. Nuclease free, PCR grade water was used as a negative control and DNA isolated from *L. donovani* was used as a positive control.

#### Agarose gel electrophoresis

Agarose powder (Agarose S; Nippon Gene Co. Ltd, Japan) was used to prepare a 2% gel with 1× TAE. A volume of 4 μl of the amplified PCR products was loaded with a 100 bp lambda marker (Invitrogen, Thermo Fisher Scientific, USA) and gel electrophoresis was performed at 100 V for 25 min. The loaded gel was stained with ethidium bromide (0.5 μg/ml) for 30 min. The migrated DNA was visualized and photographed under UV illumination.

### Statistical analysis

The data were double-checked and verified for completeness and consistency and then entered into Microsoft Access® data sheets (Version, 2013) by trained personnel while adhering to quality control procedures. Discrepant data were checked against original data forms and any mistakes were promptly corrected. The binary logistic regression was used to identify the significant socio-economic risk factors associated with CL incidence and to calculate the odds ratio (OR) along with 95% confidence intervals of the OR for each risk factor. The Chi-square test of independence was used to evaluate the significance among lesion characteristics and treatment seeking behaviour of patients with CL and to evaluate the performance of different diagnostic methods for detection of leishmaniasis. The patients were considered as positive for leishmaniasis if the specimen was positive by any of the techniques or diagnostic methods. Statistical analysis was performed using SPSS package 23.0.

## Results

### Sociodemographic risk factors

A total of 5000 individuals (4577 males and 423 females) were screened for lesions. Of them, 74 individuals who had lesions suggestive of CL were referred for further investigation. Based on laboratory investigations, a diagnosis of CL was made in 50 of these 74 personnel (67.6%).

Sociodemographic characteristics of the 50 individuals with a confirmed diagnosis of CL and the 24 individuals who had lesions suggestive of CL, but were negative by laboratory confirmation, are summarized in Table 1. Forty-eight of the 71 males (67.6%) and two of the three females (66.6%) with suspected lesions, were positive for *Leishmania* amastigotes. Even though a majority of the study population was confirmed with CL, the effect of gender on the incidence of leishmaniasis was non-significant (*P* = 0.08) at a 95% confidence level. A significantly elevated odds for the occurrence of leishmaniasis was observed in the 26–35-year age group (OR: 4.83, 95% CI: 3.49–6.20) as compared to the age group of < 25 years-old. The highest odds ratio was reported amongst soldiers whose home town was in the Southern Province of Sri Lanka (OR: 2.14, 95% CI: 1.49–4.68), while the lowest was observed from soldiers whose permanent residence was in the Western Province (OR: 0.21, 95% CI: 0.08–0.53). However, the province where the hometown is located was not identified as a significant risk factor for the prevalence of leishmaniasis incidence (*P* = 0.442).

**Table 1:** Socio-demographic factors of the study population

Factor	Response category	No. of respondents	Percentage of respondents		*P*-value	OR	95% CI
			Positive for leishmaniasis	Negative for leishmaniasis			
Gender	Male	71	67.6 (*n* = 48)	32.4 (*n* = 23)	0.080	1.00	Reference
	Female	3	66.6 (*n* = 2)	33.4 (*n* = 1)		1.04	0.96–3.49
Age (years)	< 25	12	41.7 (*n* = 5)	58.3 (*n* = 7)	0.037	1.00	Reference
	26–35	36	83.3 (*n* = 30)	16.7 (*n* = 6)		4.83	3.49–6.20
	36–45	20	60.0 (*n* = 12)	40.0 (*n* = 8)		1.75	1.27–3.16
	> 46	5	60.3 (*n* = 3)	39.7 (*n* = 2)		2.33	1.61–4.35
Province	Central	10	69.9 (*n* = 7)	30.1 (*n* = 3)	0.442	1.00	Reference
	Eastern	5	79.4 (*n* = 4)	20.6 (*n* = 1)		1.71	1.26–4.29
	North-Central	16	68.7 (*n* = 11)	31.3 (*n* = 5)		0.94	0.79–2.66
	Northern	2	50.0 (*n* = 1)	50.0 (*n* = 1)		0.43	0.15–3.51
	North-Western	9	77.9 (*n* = 7)	22.1 (*n* = 2)		1.50	1.15–3.57
	Sabaragamuwa	5	60.3 (*n* = 3)	39.7 (*n* = 2)		0.64	0.42–0.89
	Southern	6	100.0 (*n* = 6)	0 (*n* = 0)		2.14	1.49–4.68
	Uva	10	69.9 (*n* = 7)	30.1 (*n* = 3)		1.00	0.87–2.91
	Western	12	33.3 (*n* = 4)	66.7 (*n* = 8)		0.21	0.08–0.53
Education level	Grade 6–11	30	70.0 (*n* = 21)	30.0 (*n = *9)	0.360	1.00	Reference
	GCE O/L	31	58.0 (*n = *18)	42.0 (*n = *13)		0.64	0.44–1.71
	GCE A/L	12	83.3 (*n = *10)	16.7 (*n = *2)		2.14	1.49–3.85
	Graduate	1	100.0 (*n = *1)	0 (*n* = 0)		0.43	0.21–1.18
Rank	Private	24	58.3 (*n = *14)	41.7 (*n = *10)	0.625	1.00	Reference
	Lance Corporal/Corporal	37	75.6 (*n = *28)	24.4 (*n = *9)		2.22	1.54–3.33
	Sergeant/staff Sergeant/Warrant Officers	9	66.4 (*n = *6)	33.6 (*n = *3)		1.43	1.12–3.03
	2nd Lieutenant/Lieutenant/Captain	2	50.0 (*n = *1)	50.0 (*n = *1)		0.71	0.58–1.60
	Major and above	2	50.0 (*n = *1)	50.0 (*n = *1)		0.71	0.58–1.60
Unit	Infantry	35	77.2 (*n = *27)	22.8 (*n = *8)	0.235	1.00	Reference
	Support	23	52.1 (*n = *12)	47.9 (*n = *11)		0.32	0.21–1.26
	Service	14	71.4 (*n = *10)	28.6 (*n = *4)		0.74	0.49–1.54
	Sri Lanka Army Women’s Corps	2	50.0 (*n = *1)	50.0 (*n = *1)		0.30	0.23–1.08
History of working/travel in to the jungles	Yes	25	68.0 (*n = *17)	32.0 (*n = *8)	0.950	1.00	Reference
	No	49	67.4 (*n = *33)	32.6 (*n = *16)		1.03	1.00–2.06
History of overseas travel	Yes	4	75.0 (*n = *3)	25.0 (*n = *1)	0.540	1.00	Reference
	No	70	67.1 (*n = *47)	32.9 (*n = *23)		0.91	0.75–2.59
Continent of travel	Asia	2	50.0 (*n = *1)	50.0 (*n = *1)	0.710	1.00	Reference
	Out of Asia	2	50.0 (*n = *1)	50.0 (*n = *1)		1.00	0.91–3.92
	Not travelled	70	67.1 (*n = *47)	32.9 (*n = *23)		2.04	1.44–4.86
Use of insect repellant	Yes	10	40.0 (*n = *4)	60.0 (*n = *6)	0.045	1.00	Reference
	No	64	71.9 (*n = *46)	28.1 (*n = *18)		3.83	2.69–5.21
Use of mosquito nets	Yes	65	69.2 (*n = *45)	30.8 (*n = *20)	0.411	1.00	Reference
	No	9	55.7 (*n = *5)	44.3 (*n = *4)		1.80	0.99–3.22
Type of sleeves in the upper part of the uniform	Short sleeves	62	72.6 (*n = *45)	27.4 (*n = *17)	0.036	1.00	Reference
	Long sleeves	12	41.7 (*n = *5)	58.3 (*n = *7)		3.71	2.59–4.98
Length of trousers of uniform	Short trouser	14	85.7 (*n = *12)	14.3 (*n = *2)	0.107	1.00	Reference
	Long trousers	60	63.4 (*n = *38)	36.6 (*n = *22)		3.47	2.41–5.06
Footwear	Boots	25	63.9 (*n = *16)	36.1 (*n = *9)	0.27	1.00	Reference
	Slippers	27	85.2 (*n = *23)	14.8 (*n = *4)		3.23	2.24–4.57
	Does not wear foot wear regularly	4	74.5 (*n = *3)	25.5 (*n = *1)		1.69	1.04–4.09
	Shoes	28	64.3 (*n = *18)	35.7 (*n = *10)		1.01	0.97–2.14

*Notes*: The *P*-values, odds ratios and the confidence levels shown in the table are calculated by using the binary logistic regression

*Abbreviation*: OR, odds ratio; CI, confidence interval

Military personnel from lower ranks had higher exposure to CL, with the highest incidence being reported from the rank of Lance Corporal/Corporal (OR: 2.22, 95% CI: 1.54–3.33), although this was not significant. The highest number of respondents were working in the infantry regiments (*n* = 35) and of them 77.2% (*n* = 27) were positive for CL. The highest incidence of leishmaniasis was reported amongst those who did not use any insect repellent (71.9%, *n* = 46; OR: 3.83, 95% CI: 2.69–5.21) and those who wore short sleeved upper body clothing (72.6%, *n* = 45) as their uniform while attending to their duties (as compared to those who wore long sleeves) (OR: 3.71, 95% CI: 2.59–4.98). Thus, when all the sociodemographic factors were considered, only the age, wearing of short-sleeved upper clothing as a uniform and non-use of insect repellents as a protective measure were identified as significant risk factors for the occurrence of CL (Table 1).

### Lesion characteristics of the respondents

The majority of patients had a single lesion (86.0%, *n* = 43), mostly on the upper limb (67.9%, *n* = 34). None of the patients had lesions on the chest (Table 2). The location of the lesion was significantly different among the individuals who had a confirmed laboratory diagnosis of CL and suspected individuals who tested negative for *Leishmania* parasite (*χ*^2^ = 14.114, *df* = 7*, P* = 0.049). The commonest type of lesion seen in *Leishmania* positive patients were ulcerated lesions (34.0%, *n* = 17) followed by nodules (32.0%, *n* = 16), papules (28.0%, *n* = 14), and plaque lesions (6.0%, *n* = 3). Over 59.9% (*n* = 30) of the patients confirmed as CL had lesions which were about 5–10 mm diameter in size, while lesions with a diameter of 11–30 mm were also frequently found.

**Table 2:** Characteristics of lesions

Lesion features	Response category	Percentage of respondents (*n* = 74)	Percentage of respondents (*n* = 74)		*P*-value
			Positive for leishmaniasis	Negative for leishmaniasis	
Number	1	86.5 (*n = *64)	86.0 (*n = *43)	87.6 (*n = *21)	0.536
	2	8.2 (*n = *6)	10.0 (*n = *5)	4.1 (*n = *1)	
	> 2	5.4 (*n = *4)	4.0 (*n = *2)	8.3 (*n = *2)	
Site/s	Upper limb	66.2 (*n = *49)	67.9 (*n = *34)	62.6 (*n = *15)	0.049
	Lower limb	12.2 (*n = *9)	6.1 (*n = *3)	25.0 (*n = *6)	
	Face	1.4 (*n = *1)	2.0 (*n = *1)	0 (*n = *0)	
	Head	2.7 (*n = *2)	4.0 (*n = *2)	0 (*n = *0)	
	Neck	5.4 (*n = *4)	4.0 (*n = *2)	8.3 (*n = *2)	
	Chest	1.4 (*n = *1)	0 (*n = *0)	4.1 (*n = *1)	
	Back	8.1 (*n = *6)	12.0 (*n = *6)	0 (*n = *0)	
	Abdomen	2.7 (*n = *2)	4.0 (*n = *2)	0 (*n = *0)	
Type	Papule	28.4 (*n = *21)	28.0 (*n = *14)	29.1 (*n = *7)	0.946
	Nodule	33.8 (*n = *25)	32.0 (*n = *16)	37.6 (*n = *9)	
	Plaque	5.5 (*n = *4)	6.0 (*n = *3)	4.1 (*n = *1)	
	Ulcerated	32.5 (*n = *24)	34.0 (*n = *17)	29.2 (*n = *7)	
Size (mm)	< 5	5.5 (*n = *4)	2.1 (*n = *1)	12.5 (*n = *3)	0.192
	5–10	54 (*n = *40)	59.9 (*n = *30)	41.7 (*n = *10)	
	11–30	37.8 (*n = *28)	36.0 (*n = *18)	41.7 (*n = *10)	
	> 30	2.8 (*n = *2)	2.0 (*n = *1)	4.1 (*n = *1)	
Shape	Round	64.9 (*n = *48)	66.0 (*n = *33)	62.6 (*n = *15)	0.950
	Oval	31.1 (*n = *23)	30.0 (*n = *15)	33.3 (*n = *8)	
	Irregular/other	4.1 (*n = *3)	4.0 (*n = *2)	4.1 (*n = *1)	
Margin is well defined	Yes	33.8 (*n = *25)	28.0 (*n = *14)	45.9 (*n = *11)	0.048
	No	66.2 (*n = *49)	72.0 (*n = *36)	54.1 (*n = *13)	
Edge of the lesion	Regular	31.1 (*n = *23)	24.0 (*n = *12)	45.9 (*n = *11)	0.047
	Irregular	69 (*n = *51)	76.0 (*n = *38)	54.1 (*n = *13)	
Consistency	Soft	4.1 (*n = *3)	2.0 (*n = *1)	8.4 (*n = *2)	0.045
	Firm	90.6 (*n = *67)	92.0 (*n = *46)	87.5 (*n = *21)	
	Hard	5.5 (*n = *4)	6.0 (*n = *3)	4.1 (*n = *1)	
Scaling	Yes	44.6 (*n = *33)	40.1 (*n = *20)	54.3 (*n = *13)	0.251
	No	55.4 (*n = *41)	59.9 (*n = *30)	45.7 (*n = *11)	
Itching	Yes	21.7 (*n = *16)	18.1 (*n = *9)	29.1 (*n = *7)	0.047
	No	78.4 (*n = *58)	81.9 (*n = *41)	70.9 (*n = *17)	
Pain	Yes	6.8 (*n = *5)	4.0 (*n = *2)	12.6 (*n = *3)	0.046
	No	93.3 (*n = *69)	96.0 (*n = *48)	87.4 (*n = *21)	
Pus secretion	Yes	18.9 (*n = *14)	16.0 (*n = *8)	25.0 (*n = *6)	0.049
	No	81.1 (*n = *60)	84.0 (*n = *42)	75.0 (*n = *18)	
Presence of other discharge	Yes	25.7 (*n = *19)	26.0 (*n = *13)	25.0 (*n = *6)	0.927
	No	74.3 (*n = *55)	74.0 (*n = *37)	75.0 (*n = *18)	
Crusting	Yes	21.7 (*n = *16)	22.0 (*n = *11)	21.0 (*n = *5)	0.771
	No	78.4 (*n = *58)	78.0 (*n = *39)	79.0 (*n = *19)	
Skin changes	None	8.1 (*n = *6)	4.0 (*n = *2)	16.6 (*n = *6)	0.241
	Induration	37.8 (*n = *28)	40.0 (*n = *20)	33.3 (*n = *8)	
	Erythema	35.2 (*n = *26)	38.0 (*n = *19)	29.9 (*n = *7)	
	Hypopigmentation	10.8 (*n = *8)	8.0 (*n = *4)	16.6 (*n = *4)	
	Hyperpigmentation	2.7 (*n = *2)	4.0 (*n = *2)	0 (*n = *0)	
	Satellite lesion	2.7 (*n = *2)	4.0 (*n = *2)	0 (*n = *0)	
	Subcutaneous nodules	2.7 (*n = *2)	2.0 (*n = *1)	4.1 (*n = *1)	
	Scaling	0 (*n = *0)	0 (*n = *0)	0 (*n = *0)	
	Vesicles	0 (*n = *0)	0 (*n = *0)	0 (*n = *0)	

*Notes*: Suspected individuals with lesions who were confirmed to have leishmaniasis based on diagnostic tests were considered as “positive for leishmaniasis” (*n* = 50), while the rest were considered as “negative for leishmaniasis” (*n* = 24)

Lesions with poorly defined margins (72.0%, *n* = 36), irregular edges (76.0%, *n* = 38), firm consistency (92.0%, *n* = 46) and round shape (66.0%, *n* = 33) were predominant, among the CL-confirmed patients. Although a similar trend was observed with *Leishmania-*negative patients, the percentage differences among categories of nature of the margin (*χ*^2^ = 3.897, *df* = 1, *P* = 0.048), edge (*χ*^2^ = 3.935, *df* = 1, *P* = 0.047) and consistency (*χ*^2^ = 6.191, *df* = 2, *P* = 0.045) of lesions were significantly lower. Meanwhile, characteristics such as itching (81.9%, *n* = 41), pain (96.0%, *n* = 48) and pus secretion (84.0%, *n* = 42) were significantly absent among those with a confirmed diagnosis as compared to individuals in whom the disease was suspected (*χ*^2^* > *3.862, *df* = 1, *P *< 0.05). Only a few characteristics such as the site of the lesion (*χ*^2^ = 14.114, *df* = 7, *P* = 0.049), nature (*χ*^2^ = 3.897, *df* = 1, *P* = 0.048) and edge (*χ*^2^ = 3.935, *df* = 1, *P* = 0.047) of the margin and consistency (*χ*^2^ = 6.191, *df* = 2, *P* = 0.045) of the lesion, absence of itching (*χ*^2^ = 3.941, *df* = 1*, P* = 0.047) and pain (*χ*^2^ = 3.976, *df* = 1, *P* = 0.046) or pus (*χ*^2^ = 3.862, *df* = 1, *P* = 0.049) secretion denoted significant differences among the leishmaniasis positive and negative groups (Table 2).

### Treatment seeking behaviour of the respondents

The majority of laboratory-confirmed patients with CL denoted no particular treatment-seeking behaviour (38.0%, *n* = 19). However, some individuals did consult a general practitioner (24.0%, *n* = 12) or a traditional healer (20.0%, *n* = 10) as their main health-seeking behaviour or practice in response to the occurrence of a skin lesion. Similarly, those individuals who were suspected of having the disease but tested negative for CL by laboratory investigations also denoted a similar trend with lower frequencies (Table 3). A relatively higher proportion had not used any medication as a treatment option, while a variety of skin creams had been used by a selected proportion. The variations in treatment-seeking behaviour (*χ*^2^ = 3.763, *df* = 5, *P* = 0.584) or the nature of the treatment that was used (*χ*^2^ = 6.491, *df* = 3, *P* = 0.090) did not show a significant association between those who had a confirmed diagnosis of leishmaniasis and those who did not.

**Table 3:** Treatment seeking behaviour of the respondents

Factor	Response category	Percentage of respondents (*n = *74)	Percentage of respondents (*n = *74)		*P*-value
			Positive for leishmaniasis (*n = *50)	Negative for leishmaniasis (*n = *24)	
History of past health-seeking behaviour	None	36.5 (*n = *27)	38.0 (*n = *19)	33.3 (*n = *8)	0.584
	Dermatologist	6.8 (*n = *5)	6.1 (*n = *3)	8.3 (*n = *2)	
	General practitioner	27.0 (*n = *20)	24.0 (*n = *12)	33.3 (*n = *8)	
	Traditional healer	16.2 (*n = *12)	20.0 (*n = *10)	8.3 (*n = *2)	
	Military doctor	9.5 (*n = *7)	10.1 (*n = *5)	8.3 (*n = *2)	
	Other	4.1 (*n = *3)	2.1 (*n = *1)	8.3 (*n = *2)	
Type of previous treatment	None	59.5 (*n = *44)	62.0 (*n = *31)	54.3 (*n = *13)	0.090
	Cream	27 (*n = *20)	24.0 (*n = *12)	33.3 (*n = *8)	
	Oil	2.7 (*n = *2)	0 (*n = *0)	8.3 (*n = *2)	
	Other	10.9 (*n = *8)	14.1 (*n = *7)	4.3 (*n = *1)	

*Notes*: Individuals with skin lesions suspected to be CL who were confirmed to have leishmaniasis based on one or more diagnostic tests were considered as “positive for leishmaniasis” (*n* = 50), while the rest were considered as “negative for leishmaniasis” (*n* = 24)

### Performance of diagnostic methods for leishmaniasis

Amongst the 74 suspected individuals that were referred for investigations, 67.6% (*n* = 50) were confirmed to be infected with *Leishmania* by one or more microscopy-based diagnostic methods. Both SSS and TIS microscopy techniques were capable of discriminating 92.1% (*n* = 46) of the patients, along with only 12.6% (*n* = 3) of false-positive respondents, denoting a significantly higher discriminative power than LA microscopy (46.0%, *n* = 23). Meanwhile, histopathological diagnosis was confirmed only in 80.0% (*n* = 40) of the suspected individuals, showing a moderate accuracy (Table 4). Interestingly, the lowest discriminating power was shown by PCR, which resulted in only 40.0% (*n* = 20) being diagnosed as *Leishmania-*positive.

**Table 4:** Performance of the different diagnostic methods used for diagnosis of leishmaniasis

Diagnostic method	Response category	Percentage of respondents (*n* = 74)	Percentage positivity for each technique (*n* = 74)		*P*-value
			Positive (*n* = 50)	Negative (*n* = 24)	
Microscopy—slit skin smear (SSS)	Negative	31.1 (*n = *23)	4.0 (*n = *2)	87.6 (*n = *21)	< 0.001
	Positive	66.3 (*n = *49)	92.1 (*n = *46)	12.6 (*n = *3)	
	No smear/inconclusive	2.7 (*n = *2)	4.0 (*n = *2)	0 (*n = *0)	
Microscopy—lesion aspirate (LA)	Negative	64.8 (*n = *48)	48.0 (*n = *24)	99.9 (*n = *24)	< 0.001
	Positive	31.1 (*n = *23)	46.0 (*n = *23)	0 (*n = *0)	
	No smear	1.4 (*n = *1)	2.1 (*n = *1)	0 (*n = *0)	
Microscopy—tissue impression smears (TIS)	Negative	33.8 (*n = *25)	8.0 (*n = *4)	87.6 (*n = *21)	< 0.001
	Positive	66.3 (*n = *49)	92.1 (*n = *46)	12.6 (*n = *3)	
Histopathology	Negative	31.1 (*n = *23)	20.0 (*n = *10)	54.3 (*n = *13)	0.004
	Positive	69 (*n = *21)	80.0 (*n = *40)	45.9 (*n = *11)	
Polymerase chain reaction (PCR)	Negative	70.2 (*n = *52)	59.9 (*n = *30)	91.6 (*n = *22)	0.004
	Positive	29.7 (*n = *22)	40.0 (*n = *20)	8.3 (*n = *2)	

*Note*: Individuals with skin lesions suspected to be CL who were confirmed to have leishmaniasis based on one or more diagnostic tests were considered as “positive for leishmaniasis” (*n* = 50), while the rest were considered as “negative for leishmaniasis” (*n* = 24)

## Discussion

Leishmaniasis has emerged as one of the most important communicable diseases in Sri Lanka in recent years. Between the period of 2009 to 2016, nearly 8487 cases of CL have been notified representing at least one case from all 25 districts in the country. In some districts in the dry zone, the number of cases being reported is increasing with more than 100 new cases being reported each year [24, 30].

Districts in the Northern Province, which were affected by the 30-year civil conflicts now report a higher number of cases of CL due to resettlement programmes, improvement in the public health infrastructure and health care services [26]. When the patient profile is considered, a considerable number of cases recorded from these districts are military persons who reside in these areas for their employment [26]. It is well known that the occurrence of this disease is linked with age and occupation, which ultimately increases the vulnerability to receive an active vector bite [24, 31]. At the time this study commenced, it had been pointed out that there was no clear view on the disease epidemiology due to inclusion of the data regarding leishmaniasis alongside all the other patient data into the national figures [26]. Therefore, knowing the case incidence among military personnel in these areas was essential to plan disease control programmes. The clinico-epidemiological pattern of patients with CL recorded from different parts of the country have been sufficiently described and published [32–34]. To the best of our knowledge, this is the first study which has investigated the clinico-epidemiological pattern of CL amongst military personnel deployed in two previously war-torn areas of the country.

As highlighted in studies carried out for other community groups, the present investigation also reveals that it is mainly individuals within the age group of 26–35 years-old that are affected [32–36]. Therefore, this clearly indicates that people in the military are at a higher risk of exposure due to their occupation and related activities. It is known that the high incidence rate among military personnel is because they are one of the most vulnerable groups, due to continuous deployment to areas of high endemicity coupled with abundant vector populations [37, 38]. However, age cannot be considered a factor of major importance, as many of the staff members in the military belong to this age category, and personnel in older age groups may not be directly involved in outdoor activities as many of them are involved in administrative roles.

At present, the southern and northern regions of Sri Lanka report the highest disease burden [33]. Investigations highlight that a considerable proportion of patients diagnosed with leishmaniasis have a history of travel to these endemic districts. Interestingly the odds ratio of leishmaniasis infection was higher among the military personnel who indicated that their residential address belonged to the Southern Province, followed by the Eastern and North-Western provinces. The incidence rates were lower among the people who reside in non-endemic provinces such as the Western province. Therefore, it may be assumed that some of these infections may have been acquired from their residential areas rather than from the region of employment.

Preventive measures against vector biting is a key strategy in the control of transmission of vector-borne diseases [39]. This may be *via* an individual or integrated approach with different components including mechanical, chemical and biological methods being used. In leishmaniasis infections, unlike the case of mosquito bites, sand flies target skin exposed to the environment as its mouthparts are short and not strong enough to penetrate clothes [40]. This study indicated that the majority of individuals who were confirmed as leishmaniasis-positive had used short sleeve upper body uniforms. Further, only a few had used insect repellents to prevent vector bites. Therefore, these factors may have contributed positively to leishmaniasis infection among this group of military personnel. Studies conducted in Sri Lanka and selected areas in other countries (Belize and Amazon basin) have indicated that military personnel are at a greater risk because of constant and regular jungle encounters [30, 34, 38, 41]. Travel history to jungles was not a significant factor for the presence of leishmaniasis in the present study. This may be because jungle related activities of military personnel are comparatively less compared to the activities that took place during the period of the war.

A study conducted in Sri Lanka based on patient records notified during the period 2005–2015 has also emphasized a declining trend of leishmaniasis among military personnel when compared to the period of civil war in the country. This could be due to a decline in the number of soldiers being deployed in these endemic regions in addition to the fact that jungle related activities have now reduced [34]. However, the occurrence of CL among this group is still prominent due to their involvement in development projects, agriculture and farming activities which may create favourable resting and breeding habitats for sand flies, thus increasing the sand fly population [42]. On the other hand, building constructions taking place by clearing land bordering forests is a factor that results in humans being closer to the habitats of reservoir animals, which further increases the risk of infection [43].

The present study has demonstrated that single lesions are more prominent on the upper limb and that individuals were exposed to the environment as they used short sleeved upper clothing items as their attire. Further, diffuse lesions were also not found. In general, the lesion in CL starts as a classical primary lesion, subsequently progressing to generalized body rash, multiple acne form papules or hypopigmented macular lesions [33] which may lead to a misdiagnosis at the onset. However, in the current study presentation with induration and erythema of the skin or with hypopigmented lesions were uncommon. The type of lesion depends on the parasite species and immune responses in humans [44]. The papulo-ulcerative lesion has been regarded as the commonest pathological type [6, 30, 45] reported amongst army personnel [37]. The present study demonstrated that ulcerated and rounded lesion types, 3–10 mm diameter in size with no well-defined margins were more common. It was also important to note that characteristics such as itching, pain and pus secretion were significantly lacking among this group of leishmaniasis patients. Therefore, these clinical features could be used initially to characterize CL infection. Although itching is not recognized as a known feature associated with leishmanial skin lesions, studies in Sri Lanka have indicated a good minority of patients present with itching skin lesions. Hence, itchiness should not be used to exclude the cases as non-CL and laboratory confirmation is needed to confirm the diagnosis [46].

Health seeking behaviour among patients against communicable disease is a positive drive for disease control. This study has reported that the majority of patients did not seek treatment for this disease condition as they had not identified this as a significant health-related condition but as a mere skin lesion. This may be due to their negligence and lack of knowledge or awareness regarding the occurrence of skin lesions in leishmaniasis. A study conducted in the Kurunegala District of Sri Lanka has indicated that some patients sought treatment at the clinics of private health care providers in close proximity to their residence, because of the convenience in reaching the place rapidly with minimal expenditure of money and time [24]. Information regarding seeking treatment from traditional health care providers is varied. Some studies have reported that treatment has not been sought from traditional healers for a suspected lesion, but others report that more than 25% of infected patients have sought treatment from traditional healers [47, 48]. In addition, the present study identified that 13.5% of individuals who tested positive for leishmaniasis had initially consulted a traditional healer. Therefore, it is time to improve awareness and knowledge of disease aspects among this group, which is at a high risk of acquiring the disease due to their occupation. Measures have already been taken to improve their knowledge on the vector(s) (including their resting habitats, breeding habitats and peak biting hours), how lesions may present, preventive measures and available treatment options.

The present study investigated the sensitivity of different laboratory diagnostic approaches to detect CL cases. The results revealed that the microscopic examination of SSS and TIS were able to discriminate 62.2% of the patients, denoting a significantly higher discriminative power over histopathology and PCR. It is evident that smear positivity depends on the duration of the lesion, and the sensitivity of the smear significantly reduces as the lesion becomes chronic [34, 49, 50]. A similar observation was made when using PCR for diagnosis. Therefore, negative results observed in the present study over clinically suspected cases may have been due to the chronic nature of lesions that were diagnosed through the screening process after a significant duration. In the diagnosis of leishmaniasis, PCR is considered more sensitive than microscopic observations [29, 51, 52]. However, the present study reported only 27% of samples positive by PCR, which indicated the lowest sensitivity. This may be due to the low sensitivity of the LdF/LdR primer set [53] which has demonstrated only 71% sensitivity to *L. donovani* isolated among cutaneous lesions in Sri Lankan patients [54]. Therefore, it is recommended to use JW11/12 KDNA [55] or LITSR/L5.8S ITS1 primer pairs [56] as they have indicated a higher sensitivity with 100% specificity from Sri Lankan strains [54]. This study has highlighted that the selection of the LdF/LdR primer set is a limitation for PCR confirmation. A study conducted in Colombian army personnel with CL indicated that nearly 19% of samples which were positive by microscopy were negative by PCR. This may be due to potential inhibitors in the samples or unlikely manipulation of the sample. Therefore, it emphasizes the limitation of the imprint that may clarify the lack of correspondence between microscopy and PCR [37].

The main objective of the present study was to identify the clinico-epidemiological aspects of leishmaniasis transmission among military personnel, where assessment has been limited in Sri Lanka. Thus, this study was limited only to the selected at-risk community. Vector abundance in the provinces selected for the study was not assessed due to limitations in funds, resources and difficulties to conduct entomological surveys in early war-torn areas. The authors would like to highlight this as a limitation in the present study. Further, there are no detailed published studies on the prevalence of sand flies in Mullaitivu and Kilinochchi districts of Sri Lanka at present. Lack of proper documentation regarding the sand fly populations in these districts may be due to the civil war and associated security situation which existed over the past 30 years. The only published study in 2013 has evinced the presence of *Phlebotomus argentipes* predominantly in some surveyed locations in the Northern Province of Sri Lanka [56]. Therefore, *P. argentipes* could be playing the main role as the vector for leishmaniasis transmission in these areas.

In 1910, Annandale [57] reported for the first time the presence of *P. argentipes* sand fly in Sri Lanka. Since then, this species has been reported as the main vector of leishmaniasis in Sri Lanka [58]. A study conducted in the North-Western Province of Sri Lanka based on xeno-diagnostics has reported an infection rate of 2.3% among wild caught *P. argentipes* [59]. Therefore, *P. argentipes* is considered as the vector for leishmaniasis transmission in Sri Lanka.

Overall, the present study points out the occupational risk of military personnel for CL infection. Therefore, health authorities should target control efforts and programmes aimed at these occupationally high-risk groups. The Sri Lanka Army has a good public health surveillance system covering the armed services personnel deployed all over the country and leishmaniasis has been considered as a notifiable disease since 2011 in their surveillance programmes [26]. At the time this study was planned and conducted, CL cases being reported amongst the military personnel were entered into the national data base through figures of CL cases originating from the Teaching Hospital in Anuradhapura. Military personnel with suspected skin lesions were transferred to this hospital and a diagnosis was made by a consultant dermatologist. More recently, consultant dermatologists have been assigned to the general hospitals of Kilinochchi and Mullaitivu districts. It is recommended to include the figures of CL cases amongst military personnel into the national case figures as a separate entity so as to depict the real magnitude of the disease burden amongst the military in Sri Lanka.

## Conclusions

To the best of our knowledge, this study is the first comprehensive investigation on the clinical and epidemiological characteristics of CL amongst military personnel in previous war-torn areas of Sri Lanka. It is recommended to design control efforts with the involvement of public health experts in the military, as there is currently a well-established programme with specially dedicated public health inspectors to prevent infectious diseases within the military. Further, steps have been taken to improve the awareness among military personnel on preventive aspects of the disease, clinical manifestations and vector related factors. Finally, it is recommended to include the cases being reported from military personnel as a separate entity within the national figures to highlight the disease burden amongst this high-risk group in the country.

## Data Availability

Data supporting the conclusions of this article are included within the article.

## Abbreviations

CL: cutaneous leishmaniasis
FNA: fine needle aspiration
HE: hematoxylin-eosin
LA: lesion aspirate
ML: mucosal leishmaniasis
OR: odds ratio
PCR: polymerase chain reaction
SSS: slit skin smear
TIS: tissue impression smear
VL: visceral leishmaniasis
